# Does Postgraduate Education Deepen Temporomandibular Disorders Insights for Dental Professionals?

**DOI:** 10.1155/2024/3582362

**Published:** 2024-10-14

**Authors:** Zejin Liu, Jie Xiang, Yi Liu, Xueman Zhou, Yating Yi, Jun Wang, Xin Xiong

**Affiliations:** State Key Laboratory of Oral Diseases and National Center for Stomatology and National Clinical Research Center for Oral Diseases, West China Hospital of Stomatology, Sichuan University, Chengdu 610041, Sichuan, China

**Keywords:** attitudes, dental professionals, knowledge, postgraduate education, temporomandibular disorders

## Abstract

**Objective:** This study aims to investigate the impact of postgraduate education on the comprehension of Temporomandibular Disorders (TMDs) among dental professionals.

**Methods:** A cross-sectional observational study was conducted, involving 348 dental professionals, including students and practicing dentists, categorized based on their educational background into two groups: bachelor's degree or lower (Group B) and master's degree or higher (Group M). Questionnaires were utilized to assess attitudes and knowledge across four TMDs-related domains. Statistical analysis was performed to compare responses between groups and identify differences in TMDs comprehension.

**Results:** Among the 348 respondents, who participated in the study, 183 were students and 165 were practicing dentists, yielding a response rate of 79% (348/440). In the dentist group, 11 statements across every TMDs-related domain exhibited statistically significant differences in responses between Group B and Group M (*p* < 0.05). Only 2 responses from Group M conflicted with the standard answers, whereas Group B had 9 conflicting responses. In the student group, 11 statements also showed statistically significant differences between Group B and Group M (*p* < 0.05). Group M had no conflicting responses with the standard answers, while Group B had 7 conflicting responses.

**Conclusions:** Postgraduate education deepened dental professionals' understanding of TMDs. Students improved more in the domains of “diagnosis” and “treatment and prognosis,” whereas practicing dentists enhanced more in the “etiology” domain. To further advance postgraduate education, there is a need for more systematic course designs for TMDs, emphasizing the enhancement of knowledge related to examination methods and treatment options.

## 1. Introduction

Temporomandibular Disorders (TMDs) is a multifaceted condition affecting the temporomandibular joint (TMJ), masticatory muscles, and associated structures [1]. With a prevalence of approximately 30% in adults [2], it presents with a spectrum of symptoms including jaw pain, clicking or popping sounds, limited jaw movement, and headaches, leading to varying degrees of impairment in oral function and quality of life [1].

Despite its prevalence and impact, TMDs diagnosis and management remain challenging due to the heterogeneous nature of the disorder. Etiological factors encompass a wide range, including anatomical abnormalities, occlusal discrepancies, parafunctional habits, psychological stress [3], and systemic conditions such as arthritis [4]. Additionally, the subjective experience of patients also impacts TMDs management. Patients with TMDs tend to exhibit higher levels of somatic and psychological distress [5–7], which could be overlooked in the absence of pain symptoms. Current recommended practices for managing TMDs emphasize a multidisciplinary approach that includes both noninvasive and minimally invasive treatments. Guidelines recommend the use of conservative therapies such as patient education, behavioral therapy, physical therapy, and pharmacological management before considering more invasive procedure [8–10]. It is also highlighted that clinicians employ a biopsychosocial model to understand and manage the multifactorial nature of TMDs, addressing both physical and psychological aspects of the disorder [11]. This complexity emphasizes the significance of ongoing education and training to ensure that dental professionals remain well-informed and adept in managing TMDs. In China, dental education follows a structured pathway. Students typically start with a 5-year undergraduate program in stomatology, earning general dental knowledge and a bachelor's degree. For those seeking further specialization, postgraduate education is the next step, beginning with a 3-year master's program and leading to a doctorate program for advanced research and academic careers. Additionally, continuing education and professional development courses are available for practicing dentists to keep abreast of the latest developments in dental science and practice. Previous studies have found that continuing education can expose dental practitioners to new technologies and concepts, enhance their clinical skills, and change treatment practices [12, 13]. In the field of TMDs and orofacial pain, former research discovered an enhancement of dental students' understanding of TMDs and their clinical competence after undergraduate education [14–17].

Currently, numerous studies have revealed a lack of precise understanding of TMDs among dental professionals [18–29]. Reissmann et al. [18] found a lack of standardized examinations and confidence in clinical management among recently graduated dentists. Al-Huraishi et al. [19] identified a gap of understanding between newly graduated dentists and specialists regarding TMDs in the domains of “etiology” and “treatment and prognosis.” Yokoyama et al. [20] found dentists experienced distress in predicting the prognosis of TMDs treatment. These findings underscored the importance of postgraduate education and improving the current curriculum [21, 22]. However, due to the lack of research on the effect of postgraduate education on the understanding of TMDs, our knowledge of its specific impact and how to improve it remains limited.

Several factors contribute to the uncertainty surrounding the influence of postgraduate education on TMDs insights for dental professionals. First, the heterogeneity of TMDs presentations and the lack of standardized diagnostic criteria make it challenging to assess the effectiveness of educational interventions objectively [30]. Moreover, the translation of knowledge acquired into clinical practice may be influenced by individual factors such as clinical experience and practicing specialty [18, 23–25].

Therefore, the study aimed to address the following research questions: Does postgraduate education deepen TMDs insights for dental professionals? What specific domains of understanding has postgraduate education improved, and what are its shortcomings? By comparing the differences in attitude and knowledge toward TMDs between groups with different educational backgrounds, the research aims to inform the impact of postgraduate education on dental professionals' understanding of TMDs and offer valuable recommendations for future educational initiatives.

## 2. Materials and Methods

### 2.1. Participant Recruitment and Data Collection

The study adopted an observational and descriptive cross-sectional design, conducted between June 20, 2022, and March 14, 2024. Ethical approval for the study was obtained from the Research Ethics Committee at West China School of Stomatology, Sichuan University. Before filling the questionnaire, participants provided informed consent. Participants were sampled using the convenience sampling method. A total of 220 dental students and 220 practicing dentists were approached, and the questionnaire was distributed in paper format in public or private medical facilities, as well as regional dental exhibitions or dental training conferences held in Chengdu, China. Before the questionnaire distribution, participants were verbally categorized as fifth-year undergraduate interns, postgraduate students, or practicing dentists. Additionally, they were informed about the purpose of the survey to enhance their willingness to participate and to improve the response rate. Personal identification details were not requested to maintain participant anonymity. The time taken to complete the questionnaire was recorded. Additionally, a QR code linked to the online questionnaire was provided alongside the paper-based version, allowing respondents the option to complete it at their convenience. Online data collection was open until March 15, 2024. Participants who declined participation or failed to complete the questionnaire were not contacted for reminders. All involvement in the study was voluntary, and no financial compensation was offered. Exclusion criteria included: (1) failure to return the questionnaire, (2) submission of an incomplete form, or (3) completion in less than 5 min. Of the distributed questionnaires, 348 were returned, yielding a response rate of 79%.

### 2.2. Questionnaire Design and Administration

The survey comprised two sections. The first section gathered general participant information, including age, gender, and the highest level of education received (1: bachelor's degree program or lower, 2: master's degree program, or 3: doctorate program). The second section followed the questionnaire used in our previous study [25]. (SI Table 1) Comprising 25 items, the questionnaire explored attitudes towards four domains related to TMDs: “TMDs-related pain,” “etiology,” “diagnosis,” and “treatment and prognosis.” The gold standard for questionnaire answers is divided into true(T), false(F), and no consensus(N) based on references and consensus of orofacial pain specialists or TMDs specialists [1, 19, 21, 26]. Responses were recorded on a six-point scale, with options ranging from “1: strongly agree” to “5: strongly disagree,” with an additional option for “6: I don't know.” “Group consensus” was defined as more than 50% of respondents supporting either agreement or disagreement. In addition to the domains mentioned above, dentists were queried about their years of practice, type of medical facility (1: public or 2: private hospital), practicing specialty (1: general dentistry, 2: oral medicine, 3: prosthodontics, 4: oral surgery, 5: orthodontics, or 6: TMJ), and their preferred treatment approach for TMD patients (1: referral, 2: conservative treatment including hot compress, physiotherapy, and pharmacological treatment, 3: orthodontic or prosthodontic treatment, or 4: splint therapy). Additionally, dentists were asked whether they had undertaken any courses on TMDs or occlusion.

### 2.3. Sample Calculation and Statistical Analysis

The sample size was calculated using PASS2021, with *α* (two-tailed) set at 0.05 and power at 0.90. Based on the result of our previous study, the agreement ratio between groups with differing views on the same item was assumed to be 0.6 and 0.4. Maintaining a sample ratio of 1 : 1 (bachelor's degree or lower to master's degree or higher), the analysis determined that a total of 260 subjects were required for the study. Considering the recommended 60% threshold response rate for medical research, the final sample size was set at 440 subjects. Data collected from both online and offline sources were compiled using Excel software. Demographic information and the percentage distribution of answer options within each group were analyzed using SPSS software. For statistical analyses, responses indicating “strongly agree” and “agree” were grouped as “agree,” while responses indicating “strongly disagree” and “disagree” were grouped as “disagree” according to former research [19, 25, 26]. To assess differences in answer options between the two groups for each statement, the chi-square test was employed. Statistical significance was established at *p* < 0.05, indicating differences deemed noteworthy for further analysis.

## 3. Results

A total of 348 respondents completed the questionnaires, either online or offline, and were categorized based on their educational background. The group designated as “bachelor's degree program or lower” (Group B) comprised 45 males (25%) and 135 females (75%), while the “master's degree program or higher” group (Group M) consisted of 60 males (35.7%) and 108 females (64.3%). The average age of participants in each group was 31.4 ± 9.89 years and 27.12 ± 4.28 years, respectively (Table 1).

In the dental student group, 183 students were included, comprising 63 males (34.4%) and 120 females (65.60%), with an average age of 24.16 ± 2.43 years. Among the students surveyed, 66 (36.10%) were fifth-year undergraduate interns pursuing bachelor's degree and had completed their theoretical coursework, 88 (48.10%) were postgraduates enrolled in master's degree programs, and 29 (15.8%) of the students were pursuing doctorate programs (Table 2).

For the practicing dentist group, 165 dentists were surveyed, with 42 males (25.50%) and 123 females (74.50%), and an average age of 35.09 ± 8.09 years. Among these dentists, 114 (69.10%) obtained bachelor's degrees or lower, 39 (23.60%) obtained master's degrees, and 12 (7.30%) obtained doctorate degrees (Table 2). The average years of practice for dentists participating in this study were 11.62 ± 9.1, with the majority working in public hospitals (78.8%). Approximately 12.70% of surveyed dentists reported taking TMDs courses previously, while 17% claimed to have received courses on occlusion. In terms of clinical diagnosis and treatment preferences, the majority favored conservative treatment (52.10%), with referral practices constituting 38.21% when encountering patients with TMDs-related symptoms (SI Table 2). When divided by educational level, dentists in Group B were older on average and had longer working years (*p* < 0.001), and no statistical differences were observed in the rates of receiving courses on TMDs and occlusion compared with dentists in Group M (SI Table 3).

**Figure 1:**
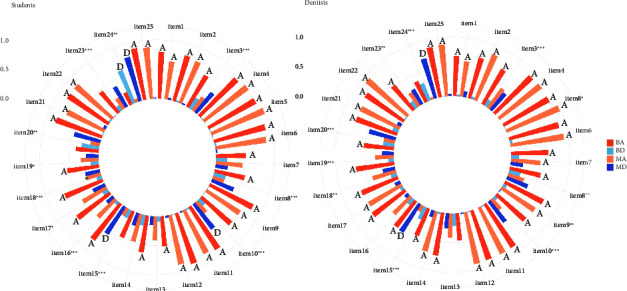
Distribution and consensus of agreement and disagreement for the 25 statements between student groups and dentist groups with different education backgrounds. BA: Agreement from the group with a Bachelor's or lower degree. BD: Disagreement from the group with a Bachelor's or lower degree. MA: Agreement from the group with a Master's or higher degree. MD: Disagreement from the group with a Master's or higher degree.

In evaluating the accuracy of the dentist group consensus, 2 responses from Group M conflicted with the standard answers, whereas Group B had 9 conflicting responses. A total of 11 statements exhibited statistically significant differences in responses between Group B and Group M (*p* < 0.05), spanning all four domains: “TMDs-related pain,” “etiology,” “diagnosis,” and “treatment and prognosis.” Notably, nine of these statements demonstrated disparate consensus levels. In these divergent views, Group B showed support for items, including “TMJ clicking is a serious symptom which often creates a painful condition,” “Nocturnal bruxism is caused by occlusal interferences,” “The position of the condyle in the fossa as seen on tomogram is a very accurate indicator of internal derangement,” “TMD is more common amongst children with mixed dentition than amongst adult with permanent dentition,” “Orthodontic treatment can prevent the onset of TMDs,” “Orthodontic treatment can treat TMDs,” “Orthodontic therapy is the best treatment to resolve TMD in a patient with a skeletal malocclusion,” and “Occlusal splints can eliminate bruxism,” on which Group M displayed varying degrees of disagreement or lack of consensus. Group B did not reach a consensus on item 24, which proposed that “All individuals with TMJ clicking need treatment,” while Group M dissented. Compared to Group B, Group M's responses were closer to the standard answer among the divergent opinions (Table 3, SI Table 4, Figure 1).

In the analysis of the student group, Group B had 7 responses that conflicted with the standard answers, while Group M had none. Student cohorts also showed statistically significant differences in responses across 11 statements (*p* < 0.05). Group M's responses were generally closer to the standard answers, except for item 16, “Measuring mouth opening capacity is a reliable assessment method,” where Group B students showed more agreement. Compared to the dentist group, where more divergent differences were observed in the “etiology” domain, students displayed greater disparities in the “diagnosis” and “treatment and prognosis” domains. Interestingly, students tended to exhibit more conservative attitudes, often indicating N or disagreement when compared to dentists in these statements (Table 4; Figure 1; SI Table 5).

## 4. Discussion

This study explored the influence of postgraduate education on the understanding of TMDs among dental professionals. Specifically, we conducted a comparative analysis of attitudes and knowledge regarding TMDs between dental professionals with different educational backgrounds (Group B vs. Group M, comprising dental students and practicing dentists, respectively). By elucidating the influence of postgraduate education on TMDs insights, this research aims to inform educational initiatives and enhance the quality of TMDs care delivery by dental professionals.

The results showed that the views presented by Group M were more aligned with existing literature and expert opinions, indicating dental professionals who have undergone postgraduate education tend to possess a better understanding of TMDs. Previous studies have also demonstrated higher levels of knowledge and clinical competence in medical professionals with advanced educational backgrounds. For instance, Vallon and Nilner [16] observed an enhancement in the perception of clinical competence in TMDs and orofacial pain by comparing odontology undergraduates and graduates. Nordin, Dawson, and Ekberg [14] and Borromeo and Trinca [31] found that as the years of education increased, dental students' knowledge of TMDs and oral-facial pain improved. Similarly, Dodds et al. [32] found enhanced performance in biomedical knowledge and clinical skill assessment among medical professionals with higher educational levels. It is expected that taking postgraduate education could potentially result in improved prevention, diagnosis, and management while also mitigating the risk of medical overuse.

The enhancement in TMDs knowledge among dental professionals can be attributed to several factors. Firstly, postgraduate programs offer clinical training in various departments and diverse theoretical curricula encompassing prosthodontics, orthodontics, occlusal lessons, etc. This aligns with the multifactorial etiology and multidisciplinary treatment of TMDs [3], which aided professionals in better understanding the disorders. Furthermore, former research found that postgraduate education fosters the development of learning capacity and strategies among medical professionals [33]. Based on this, dental professionals who underwent postgraduate education can better appraise research literature, evaluate emerging evidence, and integrate new findings into their clinical practice.

However, the research also highlights some deficiencies in postgraduate programs. Despite their higher education, professionals in Group M may still hold inaccurate opinions on specific aspects, such as the necessity of occlusal grinding treatment for TMDs and the role of mouth-opening capacity in TMDs clinical examination. A lack of consensus on these items has also been previously found in surveys of newly graduated dentists [19]. Additionally, a notable proportion of respondents exhibited uncertainty, indicating potential gaps in knowledge. This phenomenon is not uncommon in past surveys of dentists and students [20, 25, 26, 34]. These shortcomings may stem from the absence of a systematic TMDs curriculum within current postgraduate programs, potentially resulting in professionals only acquiring partial TMDs-related knowledge, leading to misunderstandings and uncertainties.

Previous studies also demonstrated that students incrementally enhance their clinical competencies and self-satisfaction in managing TMDs throughout undergraduate education [14, 15, 17]. We supposed the differences within the student group can also be attributed to the fact that students with higher education levels are older, and more clinical experience could be gained through practice, leading to a more accurate understanding. However, this trend did not manifest in the dentists' group. Group B, with relatively lower educational levels, had an older average age and longer working experience compared to Group M. Despite this, Group B demonstrated a less accurate understanding than the younger, less experienced Group M. Previous studies have also found that older age of dentists is associated with a lower understanding of TMDs [23, 24, 35]. This can be explained by the fact that Group B received their dental education earlier, and some of the TMDs treatment concepts they learned at that time are now outdated [3]. Although a previous study found that dentists who have been in practice longer tend to perform better in standardized examinations and managing TMDs, this improvement has been attributed to more opportunities for continuing education [18]. In our study, however, there was no significant difference in the rate of continuing education among doctors with different degrees.

Based on our findings, we advocate for including more comprehensive and systematically designed TMDs courses in postgraduate programs to enhance professionals' knowledge and eliminate misunderstandings, particularly in treatment modalities and clinical examination methods. For practicing dentists, more continuing education opportunities should be provided, with an emphasis on updating knowledge and eliminating clinical stereotypes. Besides offering more educational opportunities, we also advocate for dental organizations to update practicing dentists with the latest research findings and consensus through emails or notifications, thereby reducing the barriers to accessing cutting-edge knowledge in the field.

There are some limitations to our study. Firstly, certain questionnaire items lack precise answers due to the absence of evidence in evidence-based medicine, potentially impacting our assessment of respondents' knowledge gaps. Additionally, as a cross-sectional observational study, variations in respondents' exposure to education at different times and varying quality of education may confound our assessment of the impact of postgraduate education and the efficacy of the current educational system. Moreover, due to the convenience sampling method, there may be selection bias, which could impact the generalizability of the results.

In future research endeavors, we anticipate the emergence of more evidence-based studies to support precise judgments on specific TMDs-related viewpoints. We advocate for extensive, multicenter longitudinal studies to compare changes in professionals' perceptions before and after advanced education. These studies would clarify the impact of education and inform curriculum improvements. In addition, incorporating the assessment of patient care and outcomes into future studies will provide necessary insights into the practical benefits of educational interventions.

## 5. Conclusion

Postgraduate education has deepened the understanding of dental professionals regarding TMDs. Students showed greater improvement in the domains of “diagnosis” and “treatment and prognosis” while practicing dentists exhibited more enhancement in the field of “etiology.” Future postgraduate education requires more systematic course designs for TMDs, with an emphasis on enhancing evidence-based knowledge related to treatment options and examination methods.

## Figures and Tables

**Table 1:** Demographic information of the 348 participants included in the analysis.

	**Bachelor's degree or lower**	**Master's degree or higher**
*N*	180	168
Age, mean ± SD	31.4 ± 9.89	27.12 ± 4.28
Gender, *n* (%)		
Male	45 (25%)	60 (35.7%)
Female	135 (75%)	108 (64.3%)

**Table 2:** Demographic information of the students and dentists.

	**Students**	**Dentists**
*N*	183	165
Age, mean ± SD	24.16 ± 2.43	35.09 ± 8.09
Gender, *n* (%)		
Male	63 (34.40%)	42 (25.50%)
Female	120 (65.60%)	123 (74.50%)
Educational background, *n* (%)		
Bachelor's degree program or lower	66 (36.10%)	114 (69.10%)
Master's degree program	88 (48.10%)	39 (23.60%)
Doctorate program	29 (15.80%)	12 (7.30%)

**Table 3:** Significant outcomes of comparison of answer options to different statements between dentist groups with different education backgrounds.

**Item**	**Dentists with Bachelor's or lower degree**					**Dentists with Master's or higher degree**					***p***
	**Strongly agree and agree**	**Strongly disagree and disagree**	**Neutral**	**Unknown**	**Consensus**	**Strongly agree and agree**	**Strongly disagree and disagree**	**Neutral**	**Unknown**	**Consensus**	
3	78 (68.4%)^a^	14 (12.3%)^a^	21 (18.4%)^a^	1 (0.9%)^a^	A	21 (41.2%)^b^	19 (37.3%)^b^	10 (19.6%)^a^	1 (2%)^a^	N	<0.001^∗∗∗^
5	101 (88.6%)^a^	1 (0.9%)^a^	12 (10.5%)^a^	0 (0%)^a^	A	50 (98%)^b^	0 (0%)^a^	0 (0%)^b^	1 (2%)^a^	A	0.011^∗^
8	62 (54.4%)^a^	23 (20.2%)^a^	28 (24.6%)^a^	1 (0.9%)^a^	A	15 (29.4%)^b^	20 (39.2%)^b^	14 (27.5%)^a^	2 (3.9%)^a^	N	0.006^∗∗^
9	84 (73.7%)^a^	8 (7%)^a^	22 (19.3%)^a^	0 (0%)^a^	A	35 (68.6%)^a^	1 (2%)^a^	10 (19.6%)^a^	5 (9.8%)^b^	A	0.006^∗∗^
10	75 (65.8%)^a^	8 (7%)^a^	28 (24.6%)^a^	3 (2.6%)^a^	A	12 (23.5%)^b^	23 (45.1%)^b^	15 (29.4%)^a^	1 (2%)^a^	N	<0.001^∗∗∗^
15	57 (50%)^a^	26 (22.8%)^a^	28 (24.6%)^a^	3 (2.6%)^a^	A	6 (11.8%)^b^	29 (56.9%)^b^	10 (19.6%)^a^	6 (11.8%)	D	<0.001^∗∗∗^
18	77 (67.5%)^a^	12 (10.5%)^a^	25 (21.9%)^a^	0 (0%)^a^	A	21 (41.2%)^b^	11 (21.6%)^a^	19 (37.3%)^b^	0 (0%)^a^	N	0.006^∗∗^
19	75 (65.8%)^a^	11 (9.6%)^a^	27 (23.7%)^a^	1 (0.9%)^a^	A	16 (31.4%)^b^	10 (19.6%)^a^	25 (49%)^b^	0 (0%)^a^	N	<0.001^∗∗∗^
20	59 (51.8%)^a^	22 (19.3%)^a^	28 (24.6%)^a^	5 (4.4%)^a^	A	10 (19.6%)^b^	24 (47.1%)^b^	15 (29.4%)^a^	2 (3.9%)^a^	N	<0.001^∗∗∗^
23	62 (54.4%)^a^	16 (14%)^a^	33 (28.9%)^a^	3 (2.6%)^a^	A	14 (27.5%)^b^	15 (29.4%)^b^	21 (41.2%)^a^	1 (2%)^a^	N	0.005^∗∗^
24	44 (38.6%)^a^	36 (31.6%)^a^	32 (28.1%)^a^	2 (1.8%)^a^	N	2 (3.9%)^b^	36 (70.6%)^b^	13 (25.5%)^a^	0 (0%)^a^	D	<0.001^∗∗∗^

*Note:* Data are expressed as *N* (percentage). Different superscript letter indicates significant differences; without superscript or marked with same letter represent no significant differences.

Abbreviations: A, agree; N, no consensus; D, disagree.

^∗^ *p* < 0.05, ^∗∗^*p* < 0.01, and ^∗∗∗^*p* < 0.001.

**Table 4:** Significant outcomes of comparison of answer options to different statements between student groups with different education backgrounds.

**Item**	**Students with Bachelor's or lower degree**					**Students with Master's or higher degree**					***p***
	**Strongly agree and agree**	**Strongly disagree and disagree**	**Neutral**	**Unknown**	**Consensus**	**Strongly agree and agree**	**Strongly disagree and disagree**	**Neutral**	**Unknown**	**Consensus**	
3	41 (62.1%)^a^	14 (21.2%)^a^	9 (13.6%)^a^	2 (3%)^a^	A	33 (28.2%)^b^	52 (44.4%)^b^	30 (25.6%)^a^	2 (1.7%)^a^	N	<0.001^∗∗∗^
8	35 (53%)^a^	14 (21.2%)^a^	13 (19.7%)^a^	4 (6.1%)^a^	A	24 (20.5%)^b^	48 (41%)^b^	36 (30.8%)^a^	9 (7.7%)^a^	N	<0.001^∗∗∗^
10	45 (68.2%)^a^	9 (13.6%)^a^	9 (13.6%)^a^	3 (4.5%)^a^	A	29 (24.8%)^b^	63 (53.8%)^b^	17 (14.5%)^a^	8 (6.8%)^a^	D	<0.001^∗∗∗^
15	32 (48.5%)^a^	10 (15.2%)^a^	19 (28.8%)^b^	5 (7.6%)^a^	N	17 (14.5%)^b^	65 (55.6%)^b^	19 (16.2%)^b^	16 (13.7%)^a^	D	<0.001^∗∗∗^
16	51 (77.3%)^a^	4 (6.1%)^a^	10 (15.2%)^b^	1 (1.5%)^a^	A	50 (42.7%)^b^	17 (14.5%)^a^	47 (40.2%)^b^	3 (2.6%)^a^	N	<0.001^∗∗∗^
17	46 (69.7%)^a^	7 (10.6%)^a^	11 (16.7%)^a^	2 (3%)^a^	A	55 (47%)^b^	27 (23.1%)^b^	26 (22.2%)^a^	9 (7.7%)^a^	N	0.023^∗^
18	46 (69.7%)^a^	6 (9.1%)^a^	13 (19.7%)^b^	1 (1.5%)^a^	A	29 (24.8%)^b^	30 (25.6%)^b^	53 (45.3%)^b^	5 (4.3%)^a^	N	<0.001^∗∗∗^
19	36 (54.5%)^a^	9 (13.6%)^a^	19 (28.8%)^b^	2 (3%)^a^	A	35 (29.9%)^b^	25 (21.4%)^a^	53 (45.3%)^b^	4 (3.4%)^a^	N	0.011^∗^
20	26 (39.4%)^a^	20 (30.3%)^a^	16 (24.2%)^a^	4 (6.1%)^a^	N	19 (16.2%)^b^	55 (47%)^b^	32 (27.4%)^a^	11 (9.4%)^a^	N	0.006^∗∗^
23	31 (47%)^a^	7 (10.6%)^a^	24 (36.4%)^a^	4 (6.1%)^a^	N	25 (21.4%)^b^	47 (40.2%)^b^	35 (29.9%)^a^	10 (8.5%)^a^	N	<0.001^∗∗∗^
24	15 (22.7%)^a^	40 (60.6%)^a^	10 (15.2%)^a^	1 (1.5%)^a^	D	7 (6%)^b^	91 (77.8%)^b^	16 (13.7%)^a^	3 (2.6%)^a^	D	0.007^∗∗^

*Note:* Data are expressed as *N* (percentage). Different superscript letter indicates significant differences; without superscript or marked with same letter represent no significant differences.

Abbreviations: A, agree; N, no consensus; D, disagree.

^∗^ *p* < 0.05, ^∗∗^*p* < 0.01, and ^∗∗∗^*p* < 0.001.

## Data Availability

The data that support the findings of this study are available from the corresponding author upon reasonable request.

## Supporting Information

SI Table 1: twenty-five knowledge statements in four domains. SI Table 2: additional demographic information of the dentists. SI Table 3: additional demographic information of the dentists with different educational backgrounds. SI Table 4: insignificant outcomes of comparison of answer options to different statements between dentist groups with different educational backgrounds. SI Table 5: insignificant outcomes of comparison of answer options to different statements between student groups with different educational backgrounds.

## References

[1] Schiffman E., Ohrbach R., Truelove E (2014). Diagnostic Criteria for Temporomandibular Disorders (DC/TMD) for Clinical and Research Applications: Recommendations of the International RDC/TMD Consortium Network and Orofacial Pain Special Interest Group. *Journal of Oral and Facial Pain and Headache*.

[2] Valesan L. F., Da-Cas C. D., Réus J. C (2021). Prevalence of Temporomandibular Joint Disorders: A Systematic Review and Meta-Analysis. *Clinical Oral Investigations*.

[3] List T., Jensen R. H. (2017). Temporomandibular Disorders: Old Ideas and New Concepts. *Cephalalgia*.

[4] Kroese J. M., Volgenant C. M. C., Crielaard W (2021). Temporomandibular Disorders in Patients With Early Rheumatoid Arthritis and At-Risk Individuals in the Dutch Population: A Cross-Sectional Study. *RMD Open*.

[5] Felin G. C., Tagliari C. V. d C., Agostini B. A., Collares K. (2024). Prevalence of Psychological Disorders in Patients With Temporomandibular Disorders: A Systematic Review and Meta-Analysis. *The Journal of Prosthetic Dentistry*.

[6] Yap A. U., Natu V. P. (2020). Inter-Relationships Between Pain-Related Temporomandibular Disorders, Somatic and Psychological Symptoms in Asian Youths. *Journal of Oral Rehabilitation*.

[7] Yap A. U., Kim S., Lee B. M., Jo J. H., Park J. W. (2024). Correlates of Jaw Functional Limitation, Somatization and Psychological Distress Among Different Temporomandibular Disorder Diagnostic Subtypes. *Journal of Oral Rehabilitation*.

[8] Gauer R. L., Semidey M. J. (2015). Diagnosis and Treatment of Temporomandibular Disorders. *American Family Physician*.

[9] Wieckiewicz M., Boening K., Wiland P., Shiau Y. Y., Paradowska-Stolarz A. (2015). Reported Concepts for the Treatment Modalities and Pain Management of Temporomandibular Disorders. *The Journal of Headache and Pain*.

[10] Busse J. W., Casassus R., Carrasco-Labra A (2023). Management of Chronic Pain Associated with Temporomandibular Disorders: A Clinical Practice Guideline. *BMJ*.

[11] Durham J., Newton-John T. R. O., Zakrzewska J. M. (2015). Temporomandibular Disorders. *BMJ*.

[12] Eliyas S., Briggs P. F. A., Gallagher J. E. (2018). Assessing a Training Programme for Primary Care Dental Practitioners in Endodontics of Moderate Complexity: Pilot Data on Skills Enhancement and Treatment Outcomes. *British Dental Journal*.

[13] Dahlström L., Molander A., Reit C. (2015). The Impact of a Continuing Education Programme on the Adoption of Nickel-Titanium Rotary Instrumentation and Root-Filling Quality Amongst a Group of Swedish General Dental Practitioners. *European Journal of Dental Education*.

[14] Nordin S., Dawson A., Ekberg E. C. (2016). Achieved Competencies and Satisfaction in Temporomandibular Disorders and Orofacial Pain Education. *Journal of Oral and Facial Pain and Headache*.

[15] Bhagat B. R., Khairnar M. R., Maity S., Sachdev M. M., Shah S., Dharamsi R. (2024). Jaws of Knowledge: An Analysis of Temporomandibular Joint Insights in Dental Training-A Quasi-Experiment Study. *Journal of the Korean Association of Oral and Maxillofacial Surgeons*.

[16] Vallon D., Nilner M. (2009). Undergraduates’ and Graduates’ Perception of Achieved Competencies in Temporomandibular Disorders and Orofacial Pain in a Problem-Based Dental Curriculum in Sweden. *European Journal of Dental Education*.

[17] Choudhary S. H., Kale L. M., Mishra S. S., Sodhi S., Muley P., Pandey N. (2016). An Institutional Survey for Knowledge-Based and Self-Awareness Assessment in Temporomandibular Joint Disorders Among Dental Students. *Indian Journal of Dental Research*.

[18] Reissmann D. R., Behn A., Schierz O., List T., Heydecke G. (2015). Impact of Dentists’ Years since Graduation on Management of Temporomandibular Disorders. *Clinical Oral Investigations*.

[19] Al-Huraishi H. A., Meisha D. E., Algheriri W. A., Alasmari W. F., Alsuhaim A. S., Al-Khotani A. A. (2020). Newly Graduated Dentists’ Knowledge of Temporomandibular Disorders Compared to Specialists in Saudi Arabia. *BMC Oral Health*.

[20] Yokoyama Y., Kakudate N., Sumida F., Matsumoto Y., Gordan V. V., Gilbert G. H. (2018). Dentist’s Distress in the Management of Chronic Pain Control: The Example of TMD Pain in a Dental Practice-Based Research Network. *Medicine (Baltimore)*.

[21] Lindfors E., Tegelberg Å, Magnusson T., Ernberg M. (2016). Treatment of Temporomandibular Disorders—Knowledge, Attitudes and Clinical Experience Among General Practising Dentists in Sweden. *Acta Odontologica Scandinavica*.

[22] Gnauck M., Magnusson T., Ekberg E. (2017). Knowledge and Competence in Temporomandibular Disorders Among Swedish General Dental Practitioners and Dental Hygienists. *Acta Odontologica Scandinavica*.

[23] Baharvand M., Sedaghat Monfared M., Hamian M., Jalali Moghaddam E., Sadat Hosseini F., Alavi K. A. (2010). Temporomandibular Disorders: Knowledge, Attitude and Practice Among Dentists in Tehran, Iran. *Journal of Dental Research, Dental Clinics, Dental Prospects*.

[24] Mozhdeh M., Caroccia F., Moscagiuri F., Festa F., D’Attilio M. (2020). Evaluation of Knowledge Among Dentists on Symptoms and Treatments of Temporomandibular Disorders in Italy. *International Journal of Environmental Research and Public Health*.

[25] Xiong X., Xiao C., Zhou X., Li X., Wang J., Yi Y. (2023). Knowledge and Attitudes Regarding Temporomandibular Disorders Among Postgraduate Dental Students and Practicing Dentists in Western China: A Questionnaire-Based Observational Investigation. *Pain Research and Management*.

[26] Porto F., Harrell R., Fulcher R., Gonzales T. (2019). Knowledge and Beliefs Regarding Temporomandibular Disorders Among Orthodontists. *American Journal of Orthodontics and Dentofacial Orthopedics*.

[27] Osiewicz M., Kojat P., Gut M., Kazibudzka Z., Pytko-Polończyk J. (2020). Self-Perceived Dentists’ Knowledge of Temporomandibular Disorders in Krakow: A Pilot Study. *Pain Research and Management*.

[28] Tegelberg A., Wenneberg B., List T. (2007). General Practice Dentists’ Knowledge of Temporomandibular Disorders in Children and Adolescents. *European Journal of Dental Education*.

[29] López-Frías F. J., Gil-Flores J., Bonilla-Represa V., Abalos-Labruzzi C., Herrera-Martinez M. (2019). Knowledge and Management of Temporomandibular Joint Disorders by General Dentists in Spain. *Journal of Clinical and Experimental Dentistry*.

[30] Ohrbach R., Greene C. (2022). Temporomandibular Disorders: Priorities for Research and Care. *Journal of Dental Research*.

[31] Borromeo G. L., Trinca J. (2012). Understanding of Basic Concepts of Orofacial Pain Among Dental Students and a Cohort of General Dentists. *Pain Medicine*.

[32] Dodds A. E., Reid K. J., Conn J. J., Elliott S. L., McColl G. J. (2010). Comparing the Academic Performance of Graduate- and Undergraduate-Entry Medical Students. *Medical Education*.

[33] Samarakoon L., Fernando T., Rodrigo C., Rajapakse S. (2013). Learning Styles and Approaches to Learning Among Medical Undergraduates and Postgraduates. *BMC Medical Education*.

[34] De Medeiros Tormes A. K., Lemos G. A., Da Silva P. L. P (2023). Temporomandibular Disorders: Knowledge, Competency, and Attitudes of Predoctoral Dental Students. *CRANIO®*.

[35] Korkmaz Y., Candirli C., Celikoglu M., Altintas S., Coskun U., Memis S. (2016). Dentists’ Knowledge of Occlusal Splint Therapy for Bruxism and Temporomandibular Joint Disorders. *Nigerian Journal of Clinical Practice*.

