# Supplementary material for: Does Postgraduate Education Deepen Temporomandibular Disorders Insights for Dental Professionals?
Source: Pain Res Manag. 2024 Oct 14;2024:3582362. doi: 10.1155/2024/3582362 (PMC11493472; doi:10.1155/2024/3582362)
Supplement: Supporting Information — Additional supporting information can be found online in the Supporting Information section. [file 3582362.f1.docx]

**SI Table 1 Twenty-five knowledge statements in four domains**

| **Domain** | **Item** | **Statement** | **Standard answer** |
| --- | --- | --- | --- |
| **TMD-related pain** | 1 | - Sleep disturbances are common in patients with chronic orofacial pain. | T |
|  | 2 | - Depression can be an important etiologic factor in chronic orofacial pain. | T |
|  | 3 | - TMJ clicking is a serious symptom which often creates a painful condition. | F |
|  | 4 | - Oral parafunctional habits are often significant in the development of chronic TMD. | T |
| **Etiology** | 5 | - Stress is a very important factor in the development of chronic TMD. | T |
|  | 6 | - Pain is the most common reason to seek treatment of TMD. | T |
|  | 7 | - Patients with TMD who clench/brux do so either during the day or at night, but not both. | F |
|  | 8 | - Nocturnal bruxism is caused by occlusal interferences. | F |
|  | 9 | - Migraine can cause or is comorbid with facial/ jaw pain. | T |
|  | 10 | - The position of the condyle in the fossa as seen on tomogram is a very accurate indicator of internal derangement | F |
| **Diagnosis** | 11 | - Examination of neck muscles and TMJ with patients with orofacial chronic pain is important. | T |
|  | 12 | - TMD pain is aggravated/relieved by jaw motion. | N |
|  | 13 | - Reduced mouth opening capacity is almost never caused by TMJ arthritis. | F |
|  | 14 | - Palpatory tenderness in the masticatory system and/or TMJ is the most important clinical sign of TMD. | T |
|  | 15 | - TMD is more common amongst children with mixed dentition than amongst adult with permanent dentition. | F |
|  | 16 | - Measuring mouth opening capacity is a reliable assessment method. | T |
| **Treatment** **and prognosis** | 17 | - Occlusal grinding is a useful early treatment modality for TMD. | F |
|  | 18 | - Orthodontic treatment can prevent the onset of TMD. | F |
|  | 19 | - Orthodontic treatment can treat TMD. | N |
|  | 20 | - Orthodontic therapy is the best treatment to resolve TMD in a patient with a skeletal malocclusion. | F |
|  | 21 | - Anti-inflammatory drugs are effective in the treatment of acute arthralgia. | T |
|  | 22 | - The use of an occlusal splint is a good therapy in patients with TMD. | T |
|  | 23 | - Occlusal splints can eliminate bruxism. | F |
|  | 24 | - All individuals with TMJ clicking need treatment. | F |
|  | 25 | - Counselling and behavioral therapy are the first line of treatment in patients which chronic TMD. | N |

Abbreviation: TMJ: temporomandibular joint, TMD: temporomandibular disorders., T: True, F: False, N: Neutral

**SI Table 2 Additional demographic information of the dentists.**

| Year of practice, mean±SD | | 11.62±9.1 |
| --- | --- | --- |
| Medical facility, n (%) | |  |
|  | Public | 130(78.80%) |
|  | Private | 35(21.20%) |
| Department, n (%) | |  |
|  | General dentistry | 86(52.10%) |
|  | Oral medicine | 31(18.80%) |
|  | Prosthodontics | 16(9.70%) |
|  | Oral surgery | 28(17.00%) |
|  | Orthodontics | 32(19.40%) |
|  | TMJ | 3(1.80%) |
| TMD education, n (%) | | 21(12.70%) |
| Occlusal education, n (%) | | 28(17.00%) |
| Preferred treatment modality, n (%) | |  |
|  | Referral | 62(37.60%) |
|  | Conservative treatment | 86(52.10%) |
|  | Orthodontic/prosthodontic treatment | 10(6.10%) |
|  | Splint therapy | 7(4.20%) |

**SI Table 3 Additional demographic information of the dentists with different educational backgrounds.**

|  | Bachelor's degree or lower | Master's degree or higher | p |
| --- | --- | --- | --- |
| Age, n | 36.96 | 30.96 | <0.001*** |
| Year of practice, n | 14.35 | 5.62 | <0.001*** |
| TMD education, n (%) | 13(11.4%) | 8(15.7%) | 0.446 |
| Occlusal education, n (%) | 17(14.9%) | 11(24.6%) | 0.293 |

Note: *p<0.05, **p<0.01 and ***p<0.001.

**SI Table 4 Insignificant outcomes of comparison of answer options to different statements between dentist groups with different educational backgrounds.**

|  | Dentists with Bachelor's or lower degree | | | | | Dentists with Master's or higher degree | | | | | |
| --- | --- | --- | --- | --- | --- | --- | --- | --- | --- | --- | --- |
| Item | Strongly agree and agree | Strongly disagree and disagree | Neutral | Unknown | Consensus | Strongly agree and agree | Strongly disagree and disagree | Neutral | Unknown | Consensus | *p* |
| 1 | 78(68.4%) | 3(2.6%) | 26(22.8%) | 7(6.1%) | A | 31(60.8%) | 5(9.8%) | 10(19.6%) | 5(9.8%) | A | 0.19 |
| 2 | 82(71.9%) | 5(4.4%) | 24(21.1%) | 3(2.6%) | A | 43(84.3%) | 0(0%) | 6(11.8%) | 2(3.9%) | A | 0.179 |
| 4 | 101(88.6%) | 0(0%) | 13(11.4%) | 0(0%) | A | 46(90.2%) | 0(0%) | 4(7.8%) | 1(2%) | A | 0.298 |
| 6 | 100(87.7%) | 5(4.4%) | 9(7.9%) | 0(0%) | A | 47(92.2%) | 0(0%) | 2(3.9%) | 2(3.9%) | A | 0.075 |
| 7 | 72 (63.2%) | 16(14%) | 23(20.2%) | 3(2.6%) | A | 26(51%) | 11(21.6%) | 11(21.6%) | 3(5.9%) | A | 0.336 |
| 11 | 100(87.7%) | 3(2.6%) | 10(8.8%) | 1(0.9%) | A | 45(88.2%) | 0(0%) | 5(9.8%) | 1(2%) | A | 0.686 |
| 12 | 101(88.6%) | 2(1.8%) | 10(8.8%) | 1(0.9%) | A | 46(90.2%) | 1(2%) | 4(7.8%) | 0(0%) | A | 1 |
| 13 | 53(46.5%) | 24(21.1%) | 34(29.8%) | 3(2.6%) | N | 21(41.2%) | 13(25.5%) | 12(23.5%) | 5(9.8%) | N | 0.206 |
| 14 | 84(73.7%) | 4(3.5%) | 25(21.9%) | 1(0.9%) | A | 36(70.6%) | 6(11.8%) | 9(17.6%) | 0(0%) | A | 0.174 |
| 16 | 63(55.3%) | 14(12.3%) | 35(30.7%) | 2(1.8%) | A | 21(41.2%) | 4(7.8%) | 25(49%) | 1(2%) | N | 0.141 |
| 17 | 79(69.3%) | 7(6.1%) | 27(23.7%) | 1(0.9%) | A | 33(64.7%) | 5(9.8%) | 13(25.5%) | 0(0%) | A | 0.773 |
| 21 | 85(74.6%) | 12(10.5%) | 16(14%) | 1(0.9%) | A | 38(74.5%) | 4(7.8%) | 6(11.8%) | 3(5.9%) | A | 0.3 |
| 22 | 90(78.9%) | 2(1.8%) | 19(16.7%) | 3(2.6%) | A | 47(92.2%) | 0(0%) | 4(7.8%) | 0(0%) | A | 0.229 |
| 25 | 99(86.8%) | 1(0.9%) | 14(12.3%) | 0(0%) | A | 45(88.2%) | 2(3.9%) | 3(5.9%) | 1(2%) | A | 0.116 |

Note: Data are expressed as *N*(percentage). A: agree; N: no consensus; D: disagree

**SI Table 5 Insignificant outcomes of comparison of answer options to different statements between student groups with different educational backgrounds.**

|  | Students with Bachelor's or lower degree | | | | | Students with Master's or higher degree | | | | | |
| --- | --- | --- | --- | --- | --- | --- | --- | --- | --- | --- | --- |
| Item | Strongly agree and agree | Strongly disagree and disagree | Neutral | Unknown | Consensus | Strongly agree and agree | Strongly disagree and disagree | Neutral | Unknown | Consensus | *p* |
| 1 | 53(80.3%) | 0(0%) | 11(16.7%) | 2(3%) | A | 76(65%) | 4(3.4%) | 26(22.2%) | 11(9.4%) | A | 0.097 |
| 2 | 53(80.3%) | 2(3%) | 10(15.2%) | 1(1.5%) | A | 90(76.9%) | 3(2.6%) | 17(14.5%) | 7(6%) | A | 0.609 |
| 4 | 59(89.4%) | 1(1.5%) | 4(6.1%) | 2(3%) | A | 110(94%) | 0(0%) | 5(4.3%) | 2(1.7%) | A | 0.49 |
| 5 | 61(92.4%) | 1(1.5%) | 3(4.5%) | 1(1.5%) | A | 115(98.3%) | 1(0.9%) | 1(0.9%) | 0(0%) | A | 0.121 |
| 6 | 60(90.9%) | 2(3%) | 3(4.5%) | 1(1.5%) | A | 103(88%) | 4(3.4%) | 10(8.5%) | 0(0%) | A | 0.45 |
| 7 | 34(51.5%) | 13(19.7%) | 16(24.2%) | 3(4.5%) | A | 54(46.2%) | 25(21.4%) | 27(23.1%) | 11(9.4%) | N | 0.687 |
| 9 | 55(83.3%) | 1(1.5%) | 6(9.1%) | 4(6.1%) | A | 81(69.2%) | 1(0.9%) | 19(16.2%) | 16(13.7%) | A | 0.136 |
| 11 | 57(86.4%) | 2(3%) | 5(7.6%) | 2(3%) | A | 97(82.9%) | 3(2.6%) | 13(11.1%) | 4(3.4%) | A | 0.916 |
| 12 | 60(90.9%) | 1(1.5%) | 4(6.1%) | 1(1.5%) | A | 101(86.3%) | 5(4.3%) | 9(7.7%) | 2(1.7%) | A | 0.835 |
| 13 | 34(51.5%) | 6(9.1%) | 21(31.8%) | 5(7.6%) | A | 48(41%) | 18(15.4%) | 32(27.4%) | 19(16.2%) | N | 0.177 |
| 14 | 42(63.6%) | 12(18.2%) | 10(15.2%) | 2(3%) | A | 57(48.7%) | 26(22.2%) | 29(24.8%) | 5(4.3%) | A | 0.273 |
| 21 | 55(83.3%) | 1(1.5%) | 6(9.1%) | 4(6.1%) | A | 83(70.9%) | 6(5.1%) | 19(16.2%) | 9(7.7%) | A | 0.305 |
| 22 | 55(83.3%) | 2(3%) | 6(9.1%) | 3(4.5%) | A | 98(83.8%) | 1(0.9%) | 13(11.1%) | 5(4.3%) | A | 0.745 |
| 25 | 59(89.4%) | 0(0%) | 6(9.1%) | 1(1.5%) | A | 103(88%) | 2(1.7%) | 9(7.7%) | 3(2.6%) | A | 0.86 |

Note: Data are expressed as *N*(percentage). A: agree; N: no consensus; D: disagree
